# Flexible negative pressure suction ureteral access sheath combined with ureteroscopy in the treatment of upper urinary tract calculi: A meta-analysis

**DOI:** 10.1097/MD.0000000000045933

**Published:** 2025-11-21

**Authors:** Huanglin Duan, Baisheng Xu, Tianpeng Xie

**Affiliations:** a Department of Urology, The First People’s Hospital of Xiushui County, Jiujiang, Jiangxi, China; b Department of Urology, The First Affiliated Hospital of Gannan Medical University, Ganzhou, Jiangxi, China.

**Keywords:** flexible ureteral access sheath, retrograde intrarenal surgery, upper urinary tract stones, ureteroscope

## Abstract

**Background:**

Upper urinary tract stones are common diseases in urology. Ureteroscopic lithotripsy is one of the main methods for the treatment of upper urinary tract stones. With the rise of flexible urinary tract examination (F-UAS), it makes surgery safer and more effective. This study aims to compare the safety and effectiveness of flexible ureteral access sheath and traditional ureteral access sheath when used with ureteroscopic lithotripsy for treating upper urinary tract stones.

**Methods:**

A systematic search was conducted on PubMed, Embase, cochrane library, scopus, and web of science databases for studies that evaluated the use of F-UAS in treating upper urinary tract stones. The outcome measures assessed in this study included operation time, immediate stone-free rate (SFR), 1-month postoperative follow-up SFR, postoperative complication rate, postoperative fever rate, and hospital stay after surgery.

**Results:**

This study included 9 studies involving 1769 patients, with 907 in the experimental group and 862 in the control group. The meta-analysis revealed no significant difference in operation time between the experimental and control groups (MD = 4.73, 95% confidence interval [CI]: −2.41 to 11.86, *P* = .19). The experimental group demonstrated higher immediate SFR (OR = 3.84, 95% CI: 1.56–9.42, *P* = .003) and postoperative follow-up SFR (OR = 2.60, 95% CI: 1.61–4.20, *P* < .0001). Additionally, the incidence of postoperative complications was lower in the experimental group (OR = 0.47, 95% CI: 0.30–0.74, *P* = .001), as was the incidence of fever (OR = 0.27, 95% CI: 0.16–0.45, *P* < .00001). Furthermore, the postoperative hospital stay was shorter for the experimental group than the control group (MD = −0.09, 95% CI: −0.16 to 0.01, *P* = .02). No significant differences were observed in stone size (MD = 0.20, 95% CI: −0.13 to 0.53, *P* = .24) and age between the 2 groups (MD = 0.30, 95% CI: −0.88 to 1.47, *P* = .62).

**Conclusion:**

Compared to traditional ureteral access sheath, applying negative pressure suction with F-UAS for treating upper urinary tract calculi has enhanced stone clearance rates, expedited patient discharge, and decreased postoperative complications. Consequently, F-UAS may represent a superior option for retrograde internal surgery in clinical practice.

## 1. Introduction

Upper urinary tract stones are a prevalent condition in the field of urology. A study conducted in 2017 revealed that the occurrence of kidney stones alone ranges from 7% to 13% in North America, 5% to 9% in Europe, and 1% to 5% in Asia. Epidemiological surveys conducted in different regions of China indicate that the prevalence of kidney stones varies from 1.36% to 13.69%, with a noticeable upward trend.^[1]^ Both the American Urological Association and the European Urological Association guidelines recommend retrograde intrarenal surgery (RIRS) as the first-line treatment for upper urinary tract stones <20 mm.^[2,3]^ The utilize the UAS has been widespread since its introduction in 1974.^[4]^ During RIRS for the treatment of upper urinary tract stones, it offers several benefits such as improved visibility, controlled intrarenal pressure, and the ability to pass the ureteroscope multiple times to minimize damage.^[5]^ It has also been found to reduce the risk of postoperative infectious complications.^[6]^ The traditional ureteral access sheath (T-UAS) has limitations in surgical operations, for example, continuous irrigation is required when lithotripsy is performed, and poor drainage will lead to an increase in intrarenal pressure; the accumulation of intraoperative gravel pieces will lead to the phenomenon of “blizzard”; the treatment of large stones may also lead to the formation of stone streets.^[7]^ These difficulties can result in surgical complications and postoperative complications. In recent years, flexible ureteral access sheath (F-UAS) has been studied and applied as a potential solution. The F-UAS end is bendable and has an interface for connecting negative pressure suction, which can overcome some of the challenges T-UAS poses.^[7]^ Chen et al conducted a study in pig kidneys and found that the F-UAS can follow the flexible ureteroscope (F-URS) into the renal pelvis and calyces through the ureteropelvic junction, combined with negative pressure suction. Effectively avoiding interference with intrarenal pressure (IRP) control. It can also actively control IRP based on the speed of the perfusate.^[8]^ Another case report by Yue et al exhibited that a F-UAS helps maintain a clear surgical field of view.^[9]^ Due to the vacuum tube combined with negative pressure suction, the stones can be removed in time during the operation, which will effectively deal with the problems encountered by the T-UAS. In this study, we aimed to evaluate the safety and efficacy of a F-UAS for treating upper urinary tract stones.

## 2. Materials and methods

This study’s methods were conducted per the Preferred Reporting Items for Systematic Reviews and Meta-Analyses (PRISMA) statement.^[10]^ The study protocol was registered on the International Prospective Register of Systematic Reviews (PROSPERO; Registration Number: CRD420250591615).

## 3. Methods

### 3.1. Literature search

We searched the English databases PubMed, Embase, cochrane library, scopus, and web of science core collection databases. The search deadline is February 10, 2025. The search terms are “flexible vacuum-assisted UAS,” “flexible UAS”,” omni-directional UAS,” “tip bendable suction UAS”, “novel flexible UAS,” “Upper Urinary Tract Calculi”; “Upper Urinary Tract Calculi”; “Upper urinary calculi”; “renal calculi”; “ureteral calculi.” See Supplementary Material 1, Supplemental Digital Content, https://links.lww.com/MD/Q631 for specific retrieval strategies.

### 3.2. Selection criteria

#### 3.2.1. Inclusion criteria

Research in the human body; utilize a flexible access ureteral sheath to treat upper urinary tract calculi; outcome evaluation indicators included operation time, stone clearance rate, and incidence of postoperative complications.

#### 3.2.2. Exclusion criteria

Case reports, laboratory studies, review articles, animal experiments, and letters; single arm test; studies with incomplete or unavailable outcome evaluation indicators; the control group used a nontraditional UAS.

### 3.3. Screening and data extraction

Two researchers independently screened the literature according to the inclusion and exclusion criteria. Initially, the researchers eliminated literature that did not meet the inclusion criteria based on the title and abstract. Subsequently, the remaining literature was thoroughly read and screened. In cases of disagreement between the 2 researchers, a third researcher was consulted to make a final determination. The final review included the following information from the literature: title, first author, and publication time; basic characteristics of research subjects such as sample size, gender, and age; key elements of bias risk assessment; outcome indicators and corresponding result data. Ultimately, the 2 researchers selected 9 articles from the retrieved literature based on predefined inclusion and exclusion criteria, reaching a consensus on data extraction and quality evaluation.

### 3.4. Quality assessment

The Oxford centre for evidence-based medicine provides criteria for grading the level of evidence for each included study. The Newcastle–Ottawa scale (NOS) for nonrandomized controlled trials was utilized to assess the methodological quality of the investigations.^[11]^ The scoring system of this scale comprises three components: subject selection (0–4 points), comparability of study groups (0–2 points), and exposure (for case–control studies) or outcome (for cohort studies; 0–3 points). The maximum score on the NOS for any publication is 9 points (http://www.ohri.ca/programs/clinical_epidemiology/oxford.asp). A total score of ≤5 points indicates low quality, a score of 5 to 7 points indicates medium quality, and a score of >7 points indicates high quality.

The full texts of the included literature were read and independently assessed by 2 researchers. If the assessment results of 2 researchers were inconsistent, the third person performed a reassessment. Finally, the 8 articles we included were all 8 points, and the NOS score was high quality. Table 1 summarizes the risk of bias of all included retrospective studies.

**Table 1:** Newcastle–Ottawa quality assessment scale: case control studies.

Study	Selection				Comparability	Exposure			Total score
	Is the case definition adequate?	Representativeness of the cases	Selection of controls	Definition of controls	Comparability of cases and controls on the basis of the design or analysis	Ascertainment of exposure	Same method of ascertainment for cases and controls	Non-response rate	
Hua Chen 2024	☆	☆	☆	☆	☆	☆	☆	☆	8
Jie Ding 2023	☆	☆	☆	☆	☆	☆	☆	☆	8
Junkai Huang 2023	☆	☆	☆	☆	☆	☆	☆	☆	8
Yue Yu 2024	☆	☆	☆	☆	☆	☆	☆	☆	8
Zhaolin Zhang 2023	☆	☆	☆	☆	☆	☆	☆	☆	8
Zhaoxin Ying 2024	☆	☆	☆	☆	☆	☆	☆	☆	8
Hai Chang 2023	☆	☆	☆	☆	☆	☆	☆	☆	8
Jian Feng Lin 2023	☆	☆	☆	☆	☆	☆	☆	☆	8
Mehmet Uslu 2024	☆	☆	☆	☆	☆	☆	☆	☆	8

☆ means 1 point.

### 3.5. Statistical analysis

RevMan 5.3 software was utilized for data analysis. Continuous variables were represented using mean difference, when the data is expressed in quartiles, we first evaluate whether the data is skewed by Shi et al.^[12]^ If the data is normal distributioned, we estimate the mean ± standard deviation according to Luo et al^[13]^ and Wan et al.^[14]^ If the data does not belong to the normal distribution, we do not analyze this group of data. Binary variables were represented using odds ratio with a 95% confidence interval (CI). The heterogeneity between groups was assessed using the *I*² test. If the *P*-value was ≥.1 and/or the *I*² value was ≤50%, it indicated low heterogeneity, and the fixed effect model was employed for analysis. Conversely, if the heterogeneity between groups was substantial, further exploration of the heterogeneity was conducted. If heterogeneity could not be resolved, a random effects model was applied. A statistically significant difference was defined as a *P*-value <.05.

## 4. Results

### 4.1. Literature search results

**Figure 1.**
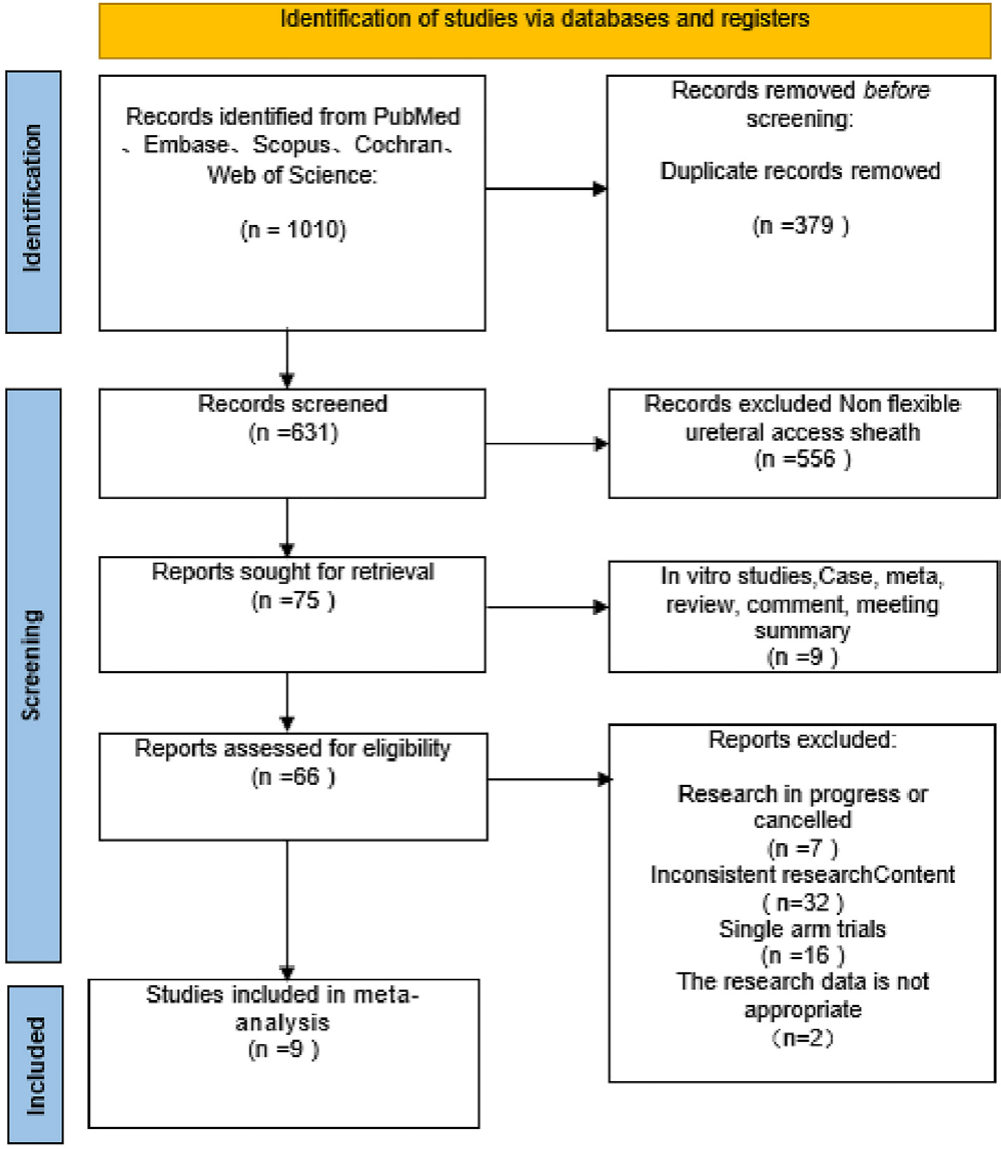
Flow diagram of study selection in the meta-analysis.

A total of 1010 relevant articles were initially screened. After excluding duplications, non-flexible UAS-related studies, in vitro experiments, and other literature, 9 studies with 1769 patients were finally obtained.^[15–23]^ The literature screening process is shown in Figure 1.

### 4.2. Study characteristics

Table 2 presents the characteristics of the 9 literatures included in the meta-analysis. All of these studies are controlled studies investigating the combination of flexible negative pressure suction UAS and ureteroscopy to treat upper urinary tract stones. The records of outcome indicators may vary, but they typically include at least 3 of the following 6 indicators: operation time, immediate stone-free rate (SFR), postoperative follow-up SFR, postoperative complication rate (CR), postoperative fever rate, and hospital stay after operation.

**Table 2:** Characteristics of included studies.

Author	Year	Research method	Intervention	Group	Gender (cases)	Age (years, mean ± standard deviation)	Stone size (mm, mean ± standard deviation)	Outcome indicators
					male/female			
Hua Chen^[15]^	2024	RR	Flexible ureteroscope	Experimental group	76/49	45.62 ± 12.93	28.1 ± 15.6	①②③④⑤⑥
				Control group	65/48	46.35 ± 14.88	26.8 ± 14.2	
Jie Ding^[16]^	2023	RR	Flexible ureteroscope	Experimental group	89/49	57.6 ± 13.7	13.0 ± 6.9	①③⑤
				Control group	39/22	55.7 ± 13.1	13.4 ± 5.2	
Junkai Huang^[17]^	2023	RR	Flexible ureteroscope	Experimental group	71/32	54.5 ± 11.0	17 ± 6	①②③④
				Control group	68/34	54.7 ± 10.7	17 ± 5	
Yue Yu^[18]^	2024	RR	Flexible ureteroscope	Experimental group	75/77	51.1 ± 12.2	15.5 ± 2.0	①②③④⑤⑥
				Control group	80/72	50.5 ± 11.8	15.2 ± 1.9	
Zhaolin Zhang^[19]^	2023	RR	Flexible ureteroscope	Experimental group	55/47	47.69 ± 9.18	18.47 ± 4.67	①②③④⑤
				Control group	71/41	46.75 ± 11.87	18.20 ± 4.46	
Zhaoxin Ying^[20]^	2024	RR	Flexible ureteroscope	Experimental group	65/38	53.08 ± 12.99	15.5 ± 5.8	①②③④⑤⑥
				Control group	103/35	53.92 ± 14.75	15.6 ± 6.4	
Hai Chang^[21]^	2023	RR	Flexible ureteroscope	Experimental group	30/14	53.82 ± 13.18	36.18 ± 5.03	①③⑤⑥
				Control group	37/15	51.20 ± 13.42	35.56 ± 5.15	
JianFeng Lin^[22]^	2023	RR	Flexible ureteroscope	Experimental group	60/37	46.8 ± 12.6	21 ± 2	①②③④⑤⑥
				Control group	53/33	47.8 ± 12.1	21 ± 3	
Mehmet Uslu^[23]^	2024	RR	Flexible ureteroscope	Experimental group	29/14	56(39, 69)	10 (8, 17)	③④⑤⑥
				Control group	27/18	55(42, 61)	11(8, 13)	

① Operation time; ② immediate stone free rate; ③ postoperative follow-up stone free rate; ④ postoperative complication rate; ⑤ postoperative fever rate;⑥ hospital stay after operation.

RR = retrospective study.

### 4.3. Study outcome

#### 4.3.1. Comparison of operation time

**Figure 2.**
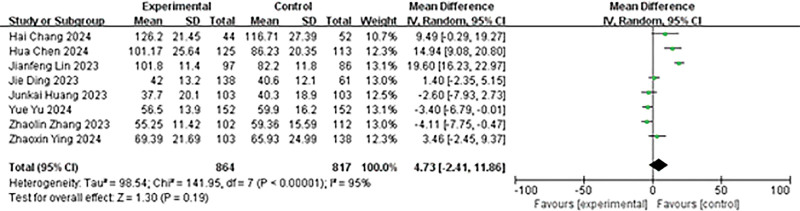
Forest plot of operation time.

A total of 8 studies reported on the operation time. The results of the heterogeneity analysis were as follows: *P* < .00001, *I*² = 95%. The random effects model was employed for the analysis. The results indicated no significant statistical difference in the operation time between the 2 groups (MD = 4.73, 95% CI: −2.41 to 11.86, *P* = .19; Fig. 2).

#### 4.3.2. Comparison of immediate SFR

**Figure 3.**
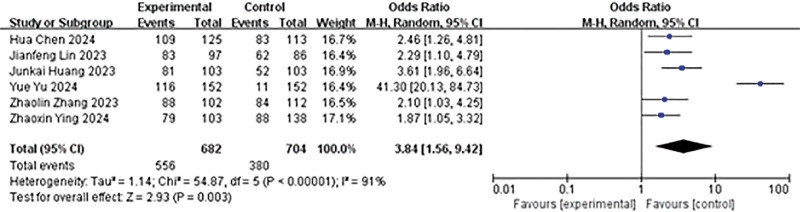
Forest plot of immediate SFR. SFR = stone-free rate.

A total of 6 studies reported on the immediate SFR. Heterogeneity analysis results were *P* < .00001, *I*² = 91%, analyzed using a random effects model. The findings indicated that the immediate stone clearance rate in the experimental group was higher than that in the control group (OR = 3.84, 95% CI: 1.56–9.42, *P* = .003; Fig. 3).

#### 4.3.3. Comparison of the postoperative follow-up SFR

**Figure 4.**
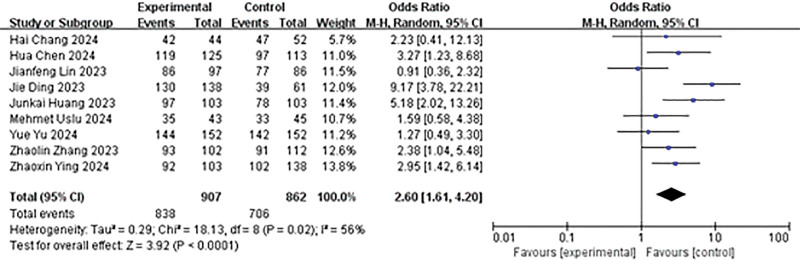
Forest plot of postoperative follow-up SFR. SFR = immediate stone-free rate.

Comparison of the SFR 1 month after surgery was reported in 9 studies in the literature. Heterogeneity analysis revealed results of *P* = .02 and *I*² = 56%, leading to the utilization of a random effects model for analysis. The findings indicated that the postoperative follow-up SFR in the experimental group was higher than in the control group (OR = 2.60, 95% CI: 1.61–4.20, *P* < .0001; Fig. 4).

#### 4.3.4. Comparison of the incidence of postoperative complications

**Figure 5.**
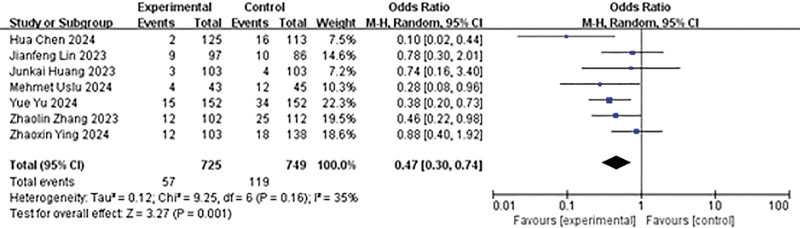
Forest plot of the incidence of postoperative complications.

A total of 7 studies reported on the incidence of postoperative complications. The results of the heterogeneity analysis exhibited a *P*-value of .16 and an *I*² value of 35%. These results were analyzed using a random effects model. The findings indicated that the experimental group exhibited a lower postoperative CR (OR = 0.47, 95% CI: 0.30–0.74, *P* = .001; Fig. 5).

#### 4.3.5. Comparison of postoperative fever rate

**Figure 6.**
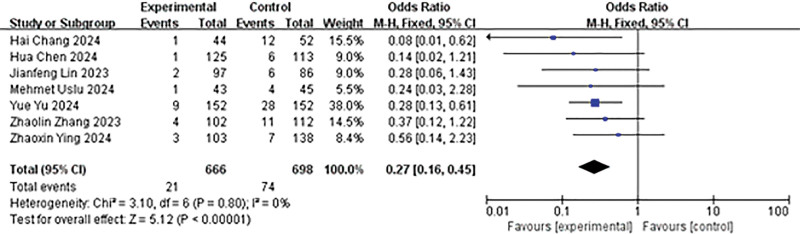
Forest plot of postoperative fever rate.

A total of 7 studies reported the incidence of postoperative fever. The heterogeneity analysis yielded the following results: *P* = .80, *I*² = 0%. The fixed effects model was employed for the analysis. The results indicated that the experimental group had a lower incidence of postoperative fever than the control group (OR = 0.27, 95% CI: 0.16–0.45, *P* < .00001; Fig. 6).

#### 4.3.6. Comparison of hospital stay after operation

**Figure 7.**
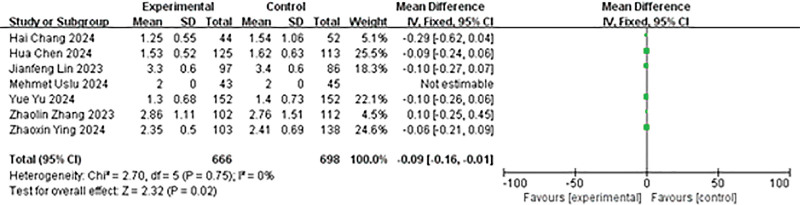
Forest plot of hospital stay after operation.

A total of 7 studies reported on the length of postoperative hospital stay. The heterogeneity analysis yielded the following results: *P* = .75, *I*² = 0%. We opted to utilize a fixed-effects model for analysis, demonstrating that the experimental group had a relatively shorter hospital stay than the control group (MD = −0.09, 95% CI: −0.16 to 0.01, *P* = .02; Fig. 7).

#### 4.3.7. Comparison of stone size

**Figure 8.**
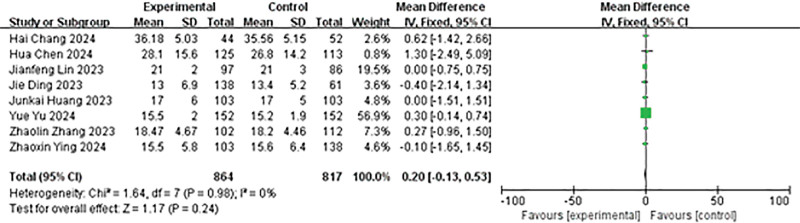
Forest plot of stone size.

One of the 9 included studies^[23]^ reported stone size using quartile data. According to Shi et al,^[12]^ they exhibited skewed analysis in stone size data. Therefore, we only statistically analyze the remaining 8 articles. The heterogeneity analysis results were: *P* = .98, *I*² = 0%, and were analyzed using the fixed effects model. The results showed no significant statistical difference in stone size between the 2 groups (MD = 0.20, 95% CI: −0.13 to 0.53, *P* = .24; Fig. 8).

#### 4.3.8. Age comparison

**Figure 9.**
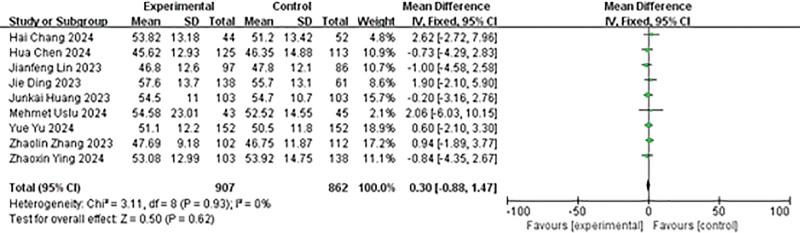
Forest plot of age.

The nine studies in the meta-analysis reported the age of all patients in the experimental and control groups. We analyzed the data of all patients. First, the heterogeneity test results exhibited that *P* = .93, *I*² = 0%, so we utilized the fixed effect model to meta-analyze the age. The results showed no significant difference in age between the experimental group and the control group (MD = 0.30, 95% CI: −0.88 to 1.47, *P* = .62; Fig. 9).

#### 4.3.9. Sensitivity analysis

**Figure 10.**
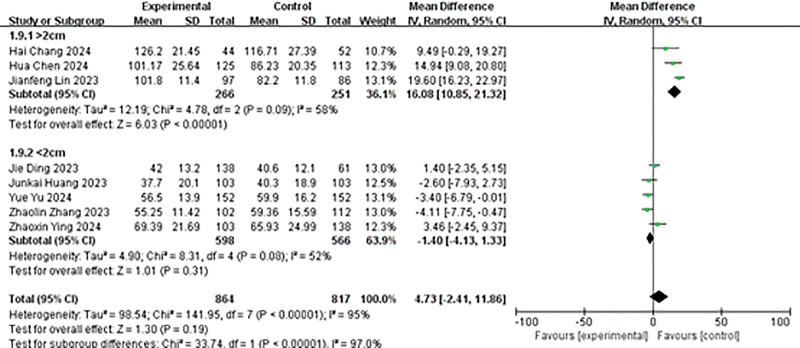
Subgroup analysis forest plot of operation time.

Heterogeneity test results showed that the studies included in the meta-analysis had significant heterogeneity in operation time and immediate stone clearance rate. Regarding operation time, we divided the stone size by 2 cm and analyzed the subgroups of >2 cm and <2 cm. Although there was still heterogeneity, it was significantly reduced (>2 cm group *P* = .09, *I*² = 58%; <2 cm group *P* = .08, *I*² = 52%). The results of meta-analysis showed that the operation time of the experimental group was longer than that of the control group in the >2 cm group (MD = 16.08, 95% CI: 10.85–21.32, *P* < .00001; Fig. 10), while there was no significant difference in the operation time between the experimental group and the control group in the <2 cm group (MD = −1.40, 95% CI: −4.13 to 11.33, *P* = .31; Fig. 10).

**Figure 11.**
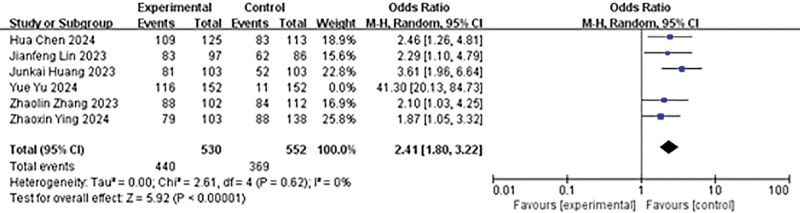
Subgroup analysis forest plot of immediate SFR. SFR = stone-free rate.

In the sensitivity analysis of the immediate SFR, we eliminated a result with significant bias due to publication bias. This exclusion significantly reduced heterogeneity (*P* = .62, *I*² = 0%), and the results were consistent with those before exclusion (OR = 2.41, 95% CI: 1.80–3.22, *P* < .00001; Fig. 11).

## 5. Discussion

The current treatment methods for upper urinary tract stones primarily consist of extracorporeal shock wave lithotripsy, laparoscopic incisional lithotomy, percutaneous nephrolithotomy, and ureteroscopic lithotripsy.^[24]^ According to the American Urological Association guidelines, medical expulsive therapy, such as α-receptor blockers, should be administered for stones smaller than 10 mm. extracorporeal shock wave lithotripsy is associated with the lowest CR for upper urinary tract stones when comparing treatment options. However, RIRS demonstrates the highest stone clearance rate. For middle and lower ureteral stones requiring intervention, RIRS should be prioritized as the first-line treatment. This recommendation also applies to renal stones measuring 20 mm or less. Conversely, percutaneous nephrolithotomy (PCNL) remains the first-line treatment for renal stones larger than 20 mm.^[2]^ There are few indications for laparoscopic or open surgery, primarily reserved for cases where PCNL puncture or the RIRS approach proves unsuccessful.^[3]^ The meta-analysis results of the study by Kang et al demonstrated that the stone clearance rate of RIRS in the treatment of kidney stones measuring 20 to 35 mm ranged from 71% to 95%.^[25]^ RIRS facilitates the transition from artificial channels to natural channels. This surgical technique minimizes patient trauma, establishing itself as a safer and more minimally invasive option for the treatment of upper urinary tract calculi.^[26]^ Interestingly, many patients prefer undergoing multiple RIRS procedures over PCNL when selecting their surgical options.

Although RIRS is widely used to treat upper urinary tract calculi, the presence of residual stones post-surgery remains a significant challenge. Rippe et al^[27]^ conducted computed tomography scans on 256 patients treated with RIRS 30 to 90 days postoperatively, revealing that 38% of patients had residual fragments according to computed tomography standards, with over 50% of these fragments measuring 1 cm or larger. Although we have consistently emphasized that stones <4 mm are clinically insignificant, numerous studies on postoperative follow-up have confirmed that residual stones pose a risk for future stone recurrence. Simon et al^[28]^ followed up with 85 patients and found that the presence of residual stones did not influence ipsilateral stone-related events in high-risk patients; however, in low-risk patients, 33.3% of those with residual stones experienced ipsilateral stone-related events, while none of the patients without residual stones did. They argue that residual stones of any size should not be dismissed as “no stones,” and that endoscopic stone treatment should aim to eradicate all stones. Another study^[29]^ indicated residual debris, particularly fragments larger than 4 mm, appears to elevate the risk of stone-related episodes and the likelihood of future surgical reinterventions. Kang et al^[30]^ reported that 18.1% (19/105) of patients with residual stones <1 mm and 28.6% (8/28) of patients with residual stones <3 mm exhibited complications after 2 years of follow-up. To improve SFR, novel technologies have been introduced to extract residual fragments, including a polyethylene endoscopic pouch and a biocompatible stone adhesive. However, these methods have yet to achieve complete stone clearance.^[31,32]^ Because T-UAS can not be bent, and need to undertake the task of drainage, it is often placed ureteropelvic junction, when the renal calyceal stone angle deviates from the calyceal neck, only the F-URS body can be extended, however, in this way, the stones are often easy to move, resulting in the failure of lithotripsy, and ultimately the residual stones. T-UAS can not effectively suck out stones and infuse liquid/blood during the operation. When the stone fragments accumulate during the operation, although there is no interference of blood and/or pus, it will also cause a “blizzard” phenomenon, which will still affect the lithotripsy process and lead to residual stones; when the patient’s stones are too large, it is likely to lead to the formation of postoperative stone streets, which is one of the reasons why RIRS is not recommended for large stones. However, the F-UAS can potentially overcome the limitations encountered by T-UAS. The F-UAS features a 10 cm long tube at its distal end, which exhibits excellent flexibility and deformability, allowing it to be bent in conjunction with the F-URS. The head of the sheath is equipped with 2 ports: a 45° port connected to a negative pressure suction pipeline, and a horizontal port that aligns with the ureteral access sheath (UAS) for inserting the F-URS. Importantly, as the ureteroscope is withdrawn, the negative pressure effect facilitates the removal of debris near the sheath from the body. Additionally, the F-UAS can be passively bent by the movements of the ureteroscope. When the F-UAS and F-URS are positioned on the same horizontal plane, the bending angle can reach up to 270°,^[33]^ which can enter most renal pelvis and calyces at will. The flexibility of t-uas enables it to better adapt to the anatomical structure of the kidney, especially in treating lower calyceal calculi. Studies have shown that compared with the T-UAS, F-UAS has a higher stone clearance rate and shorter operation time in the treatment of lower calyceal calculi^[34]^, it is design can also be safely used in complex anatomy (such as kidney transplantation or child patients).^[35,36]^ In addition, even when a stone is situated in the narrow neck of the calyx or the deeper segments of the calyceal body, the bending capability of the flexible sheath allows it to trap the stone effectively and effectively suck the stone out of the body. F-UAS can improve the stone clearance rate through its unique tip design and negative pressure suction function. For example, 1 study showed that the single-stage stone clearance rate using F-UAS was as high as 95.5%, and the rate of unplanned readmission was very low.^[37,38]^ This mechanism facilitates the crushing and aspiration of the stone, thereby minimizing the reliance on stone baskets. Unfortunately, only 2 of the studies in our statistical analysis reported using stone baskets, limiting our ability to conduct a comprehensive analysis. Two studies focusing on stone basket usage indicated that the F-UAS demonstrated a lower utilization rate than the T-UAS in the context of RIRS.^[18,20]^ Moreover, a global multicenter study by Zhu et al corroborated this finding.^[39]^ Reducing the use of stone baskets can lower costs for patients, thereby alleviating the financial burden associated with the global treatment of urinary calculi to some extent.

Infection and bleeding are well-documented complications associated with RIRS, occurring both intraoperatively and postoperatively. A multicenter study by Carlo et al^[40]^ revealed that 4.5% of patients experienced intraoperative bleeding from the pelvicalyceal system. Additionally, the earliest postoperative complications included fever and infection requiring antibiotic treatment in approximately 6.3% of cases, with blood transfusions needed in 5.5% and sepsis necessitating admission to the intensive care unit in 1.3%. It is crucial to note that elevated IRP is a significant risk factor for both infection and bleeding.^[41–43]^ Previous studies have demonstrated a positive correlation between the incidence of infection and IRP.^[44,45]^ To achieve an optimal surgical field, adequate irrigation flow is essential, as IRP is frequently influenced by continuous liquid irrigation. Suppose the irrigation fluid is not completely drained. In that case, the renal collection system may experience elevated pressure, which can cause infected urine to flow backward or leak into the perirenal tissue, thereby facilitating the spread of infection and potentially leading to sepsis.^[46]^ A study has demonstrated that washing with a low flow rate and low pressure, along with utilizing a large-diameter UAS, is essential for maintaining a low intrarenal pressure (IRP) to mitigate systemic inflammatory response syndrome,^[47]^ particularly when utilizing a T-UAS. Negative pressure suction can achieve a lower IRP, facilitating timely and sufficient entry of renal irrigation fluid. Currently, the application of negative pressure ureteral access sheaths in conjunction with RIRS for the treatment of renal calculi is rapidly gaining traction. Compared to traditional nonnegative pressure UASs combined with RIRS, negative pressure ureteral access sheaths has demonstrated a reduced postoperative infection rate in experienced centers.^[46]^

The most common factors inducing postoperative bleeding include the renal decompression effect, renal vascular malformations, and continuous high-pressure perfusion in the renal pelvis. Rehman et al^[48]^ conducted ureteroscopy on 7 cadaveric kidneys without the placement of a UAS. When perfusion was conducted at a pressure of 19.6 kPa, the renal pelvic pressure increased to between 5.096 and 5.782 kPa, with the maximum pressure potentially reaching physiological levels, approximately 0.978 kPa, dozens of times higher. The renal fornix represents the weakest area in the renal pelvis. When the pressure in the renal pelvis exceeds 75 mm Hg (1 mm Hg = 0.133 kPa), it may lead to renal rupture.^[49]^ The T-UAS integrates a negative pressure suction function, which regulates the pressure in the renal pelvis and reduces the risk of perfusion fluid reflux and postoperative infection. This feature is significant for treating patients with large stones (≥3 cm) or those complicated by urinary tract infections.^[50,51]^ The elevated pressure within the renal pelvis increases tension on the renal capsule. When this tension exceeds a critical threshold, a renal subcapsular hematoma can form, potentially leading to capsule rupture and significant hemorrhage. Current recommendations suggest maintaining the IRP below 30 cm H_2_O during ureteroscopic surgery to avert ureteral and renal pelvic perfusion pressure increases during the procedure.^[52]^ According to Zhong et al,^[52]^ intelligent pressure control has been implemented in RIRS. Their study demonstrated that high-flow perfusion could be sustained throughout the operation, allowing the holmium laser to continuously perform lithotripsy without inducing thermal damage to the ureter. The combination of the flexible negative pressure suction UAS and the negative pressure suction device creates a continuous perfusion-suction cycle, enhancing the outflow of intrarenal perfusion fluid and maintaining low intrarenal pressure.^[39]^ In the process of lithotripsy, due to the interference of gravel, blood, or pus, continuous liquid perfusion and irrigation are needed to maintain clear vision, which leads to the continuous increase of IRP, which may lead to an increase in infection complications and may lead to renal capsule hemorrhage and even renal rupture. During flexible negative pressure suction sheath procedures combined with ureteroscopic lithotripsy, the negative pressure suction actively removes fluid from the kidney, including pus and blood. This ensures a clear surgical field of vision and significantly reduces the risk of intrarenal hypertension associated with T-UAS.

In recent years, the introduction of F-UAS has significantly improved the gravel clearance rate in RIRS while also appearing to reduce postoperative complications. Due to its potential for enhancing effectiveness and safety, an increasing number of studies have confirmed these benefits.^[53]^ This study demonstrates that F-UAS exhibits significant advantages in various aspects. Furthermore, it is essential to note that the NOS scoring system indicates the comparability between groups, the representativeness of exposed populations, the accuracy of outcome indicators, and the reliability of follow-up records across all included studies. Consequently, our results are credible from these perspectives. However, it is essential to acknowledge that publication and selection bias may still be present, particularly in retrospective studies.

The outcome indicators reported in the 7 studies varied regarding complications, including fever, hematuria, infection, colic, stone obstruction, vomiting, and mucosal injury. We conducted a combined analysis of the causes of these complications. Most complications were primarily related to fever and bleeding, while other complications occurred infrequently.

However, when compared to laparoscopic incisional lithotomy and percutaneous nephrolithotomy, ureteroscopic lithotripsy performed via the natural orifice results in less tissue damage. Recently, it has emerged as the most common treatment method for upper urinary tract stones. The F-UAS effectively controls intrarenal pressure, reduces the incidence of postoperative complications, and enhances the stone clearance rate, thereby providing an efficient and safe treatment option for upper urinary tract stones. Our meta-analysis further substantiates this perspective.

## 6. Limitations

This meta-analysis has some limitations: all the included studies were retrospective; regarding the research characteristics of F-UAS, retrospective studies lead to incomplete data, selection bias may also exist, and imbalance between groups is also a problem; each study’s outcome indicators are inconsistent, and the inclusion of postoperative complications differs; some literature sample sizes are small; among the outcome indicators we selected for analysis, only intraoperative fever and postoperative hospital stay have low heterogeneity; the rest have medium and high heterogeneity; publication bias always exists in many studies. The loss of negative results is also huge for the actual research results.

## 7. Conclusion

The existing evidence examines the safety of combining F-UAS with F-URS for treating upper urinary tract stones. The analysis considers various factors such as operation time, immediate stone clearance rate, postoperative follow-up SFR, postoperative CR, postoperative fever rate, hospital stay after operation, and effectiveness. The results indicate that, despite no significant statistical difference in stone size, the experimental group had a higher stone clearance rate than the control group and a lower incidence of postoperative complications and fever. However, it is worth noting that the current research on F-UAS is mainly retrospective. Therefore, future studies should include more high-quality, large-sample, multicenter randomized controlled trials to provide reliable evidence.

## Author contributions

**Conceptualization:** Huanglin Duan, Tianpeng Xie.

**Data curation:** Huanglin Duan, Baisheng Xu.

**Formal analysis:** Huanglin Duan, Baisheng Xu.

**Software:** Huanglin Duan.

**Writing – original draft:** Huanglin Duan.

**Writing – review & editing:** Baisheng Xu, Tianpeng Xie.

## Supplementary Material


