# Supplementary material for: Flexible negative pressure suction ureteral access sheath combined with ureteroscopy in the treatment of upper urinary tract calculi: A meta-analysis
Source: Medicine (Baltimore). 2025 Nov 21;104(47):e45933. doi: 10.1097/MD.0000000000045933 (PMC12643778; doi:10.1097/MD.0000000000045933)
Supplement: Supplementary file 1 [file medi-104-e45933-s001.doc]

# PubMed

Search: ****(((((flexible vacuum-assisted ureteral access sheath) OR (omni-directional ureteral access sheath)) OR (flexible ureteral access sheath)) OR (tip bendable suction ureteral access sheath)) OR (novel flexible ureteral access sheath)) AND (((((((((((((((((((((((Upper urinary tract calculi) OR (Upper urinary calculi)) OR (Renal calculi)) OR (Calculi, Kidney)) OR (Calculus, Kidney)) OR (Kidney Calculus)) OR (Nephrolith)) OR (Renal Calculus)) OR (Kidney Stones)) OR (Kidney Stone)) OR (Stone, Kidney)) OR (Stones, Kidney)) OR (Renal Calculi)) OR (Calculi, Renal)) OR (Calculus, Renal)) OR (ureteral stones)) OR (ureteral calculi)) OR (Calculi, Ureteral)) OR (Calculus, Ureteral)) OR (Ureteral Calculus)) OR ("Kidney Calculi"[Mesh])) OR ("Ureteral Calculi"[Mesh])))****

# **Cochrane**


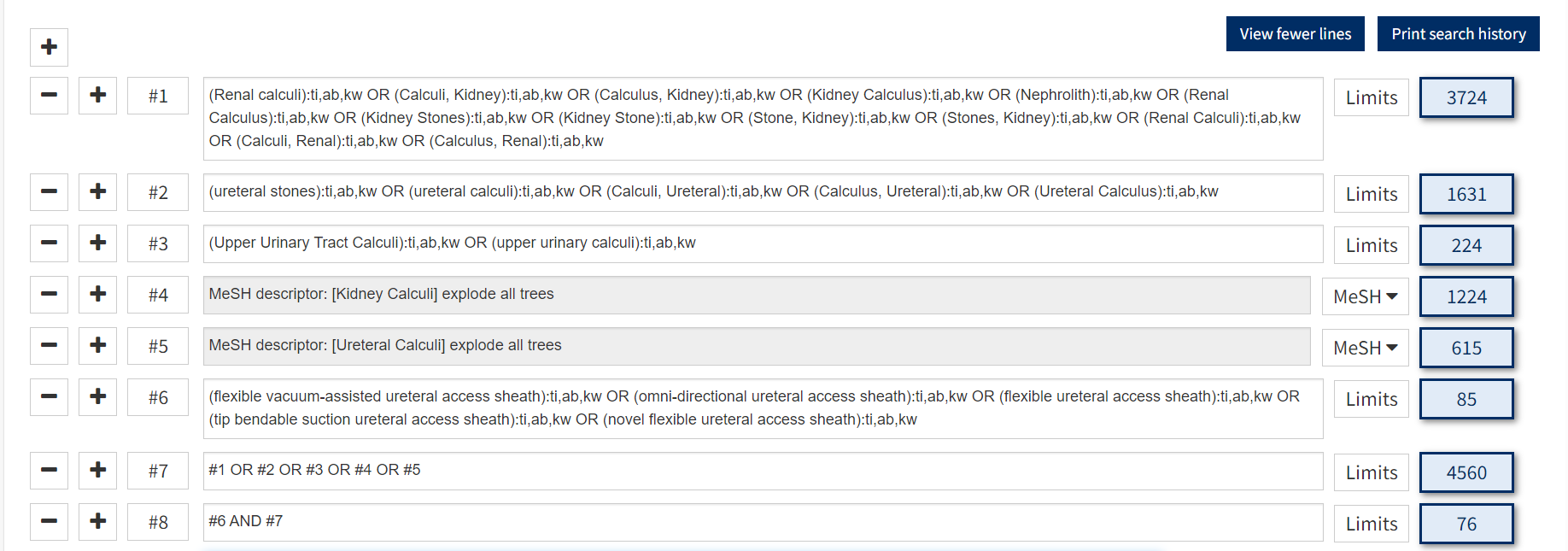


#1 (Renal calculi):ti,ab,kw OR (Calculi, Kidney):ti,ab,kw OR (Calculus, Kidney):ti,ab,kw OR (Kidney Calculus):ti,ab,kw OR (Nephrolith):ti,ab,kw OR (Renal Calculus):ti,ab,kw OR (Kidney Stones):ti,ab,kw OR (Kidney Stone):ti,ab,kw OR (Stone, Kidney):ti,ab,kw OR (Stones, Kidney):ti,ab,kw OR (Renal Calculi):ti,ab,kw OR (Calculi, Renal):ti,ab,kw OR (Calculus, Renal):ti,ab,kw 3724

#2 (ureteral stones):ti,ab,kw OR (ureteral calculi):ti,ab,kw OR (Calculi, Ureteral):ti,ab,kw OR (Calculus, Ureteral):ti,ab,kw OR (Ureteral Calculus):ti,ab,kw 1631

#3 (Upper Urinary Tract Calculi):ti,ab,kw OR (upper urinary calculi):ti,ab,kw 224

#4 MeSH descriptor: [Kidney Calculi] explode all trees 1224

#5 MeSH descriptor: [Ureteral Calculi] explode all trees 615

#6 (flexible vacuum-assisted ureteral access sheath):ti,ab,kw OR (omni-directional ureteral access sheath):ti,ab,kw OR (flexible ureteral access sheath):ti,ab,kw OR (tip bendable suction ureteral access sheath):ti,ab,kw OR (novel flexible ureteral access sheath):ti,ab,kw 85

#7 #1 OR #2 OR #3 OR #4 OR #5 4560

#8 #6 AND #7 76

# Web of science


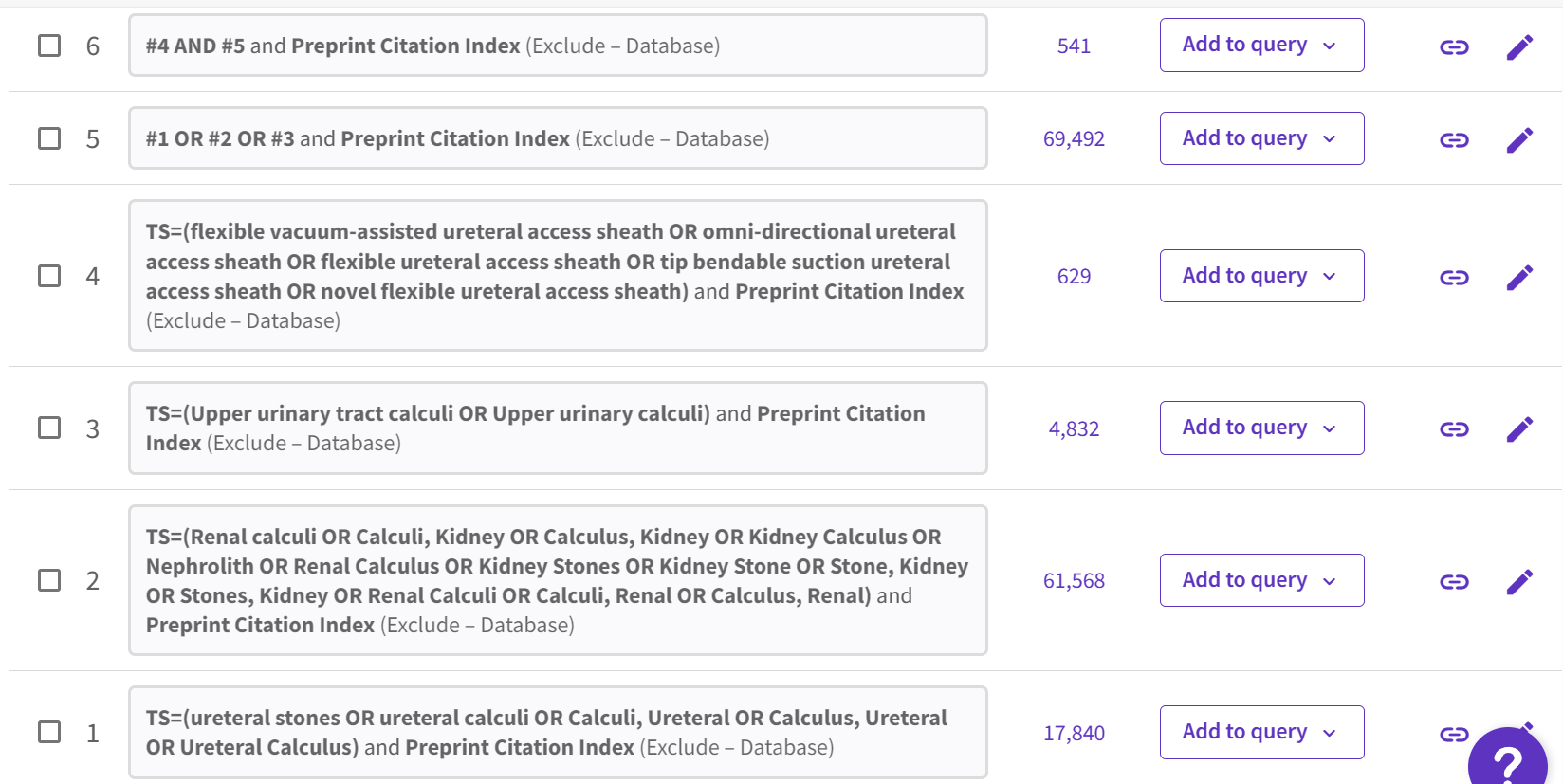


# Searches:

1: TS=(ureteral stones OR ureteral calculi OR Calculi, Ureteral OR Calculus, Ureteral OR Ureteral Calculus) and Preprint Citation Index (Exclude – Database) Date Run: Mon Feb 10 2025 20:17:12 GMT+0800 (中国标准时间) Results: 17840

2: TS=(Renal calculi OR Calculi, Kidney OR Calculus, Kidney OR Kidney Calculus OR Nephrolith OR Renal Calculus OR Kidney Stones OR Kidney Stone OR Stone, Kidney OR Stones, Kidney OR Renal Calculi OR Calculi, Renal OR Calculus, Renal) and Preprint Citation Index (Exclude – Database) Date Run: Mon Feb 10 2025 20:17:33 GMT+0800 (中国标准时间) Results: 61568

3: TS=(Upper urinary tract calculi OR Upper urinary calculi) and Preprint Citation Index (Exclude – Database) Date Run: Mon Feb 10 2025 20:18:08 GMT+0800 (中国标准时间) Results: 4832

4: TS=(flexible vacuum-assisted ureteral access sheath OR omni-directional ureteral access sheath OR flexible ureteral access sheath OR tip bendable suction ureteral access sheath OR novel flexible ureteral access sheath) and Preprint Citation Index (Exclude – Database) Date Run: Mon Feb 10 2025 20:18:31 GMT+0800 (中国标准时间) Results: 629

5: #1 OR #2 OR #3 and Preprint Citation Index (Exclude – Database) Date Run: Mon Feb 10 2025 20:19:07 GMT+0800 (中国标准时间) Results: 69492

6: #4 AND #5 and Preprint Citation Index (Exclude – Database) Date Run: Mon Feb 10 2025 20:19:36 GMT+0800 (中国标准时间) Results: 541

# Embase

Session Results

.......................................................

No. Query Results Results Date

#6. #4 AND #5 15 7 Feb 2025

#5. #1 OR #2 OR #3 25,232 7 Feb 2025

#4. 'flexible vacuum-assisted ureteral access 23 7 Feb 2025

sheath':ab,ti OR 'flexible ureteral access

sheath':ab,ti OR 'omni-directional ureteral

access sheath':ab,ti OR 'tip bendable suction

ureteral access sheath':ab,ti OR 'novel flexible

ureteral access sheath':ab,ti

#3. 'ureteral stones':ab,ti OR 'ureteral 6,503 7 Feb 2025

calculi':ab,ti OR 'calculi, ureteral':ab,ti OR

'calculus, ureteral':ab,ti OR 'ureteral

calculus':ab,ti

#2. 'calculi, kidney':ab,ti OR 'calculus, 18,966 7 Feb 2025

kidney':ab,ti OR 'kidney calculus':ab,ti OR

'nephrolith':ab,ti OR 'renal calculus':ab,ti OR

'kidney stones':ab,ti OR 'kidney stone':ab,ti OR

'stone, kidney':ab,ti OR 'stones, kidney':ab,ti

OR 'renal calculi':ab,ti OR 'calculi,

renal':ab,ti OR 'calculus, renal':ab,ti

#1. 'upper urinary tract calculi':ab,ti OR 'upper 648 7 Feb 2025

urinary calculi':ab,ti

# Scopus

( ( ALL ( "upper urinary tract calculi*" ) ) OR ( ALL ( "upper urinary calculi*" ) ) OR ( ALL ( "ureteral calculi*" ) ) OR ( ALL ( "renal calculi*" ) ) ) AND ( ( ALL ( "flexible vacuum-assisted ureteral access sheath*" ) ) OR ( ALL ( "omni-directional ureteral access sheath*" ) ) OR ( ALL ( "flexible ureteral access sheath*" ) ) OR ( ALL ( "tip bendable suction ureteral access sheath*" ) ) OR ( ALL ( "novel flexible ureteral access sheath*" ) ) )
